# Small Bowel Obstruction After Neonatal Repair of Congenital Diaphragmatic Hernia—Incidence and Risk-Factors Identified in a Large Longitudinal Cohort-Study

**DOI:** 10.3389/fped.2022.846630

**Published:** 2022-05-17

**Authors:** Katrin B. Zahn, Anna-Maria Franz, Thomas Schaible, Neysan Rafat, Sylvia Büttner, Michael Boettcher, Lucas M. Wessel

**Affiliations:** ^1^Department of Pediatric Surgery, University Children's Hospital Mannheim, University of Heidelberg, Mannheim, Germany; ^2^ERNICA Centre, Mannheim, Germany; ^3^Department of Neonatology, University Children's Hospital Mannheim, University of Heidelberg, Mannheim, Germany; ^4^Department of Medical Statistics and Biomathematics, Medical Faculty Mannheim, University of Heidelberg, Mannheim, Germany

**Keywords:** longitudinal follow-up, intestinal complications, congenital diaphragmatic hernia (CDH), adhesions, adhesive small bowel obstruction (SBO), risk factors

## Abstract

**Objective:**

In patients with a congenital diaphragmatic hernia (CDH), postoperative small bowel obstruction (SBO) is a life-threatening event. Literature reports an incidence of SBO of 20% and an association with patch repair and ECMO treatment. Adhesions develop due to peritoneal damage and underly various biochemical and cellular processes. This longitudinal cohort study is aimed at identifying the incidence of SBO and the risk factors of surgical, pre-, and postoperative treatment.

**Methods:**

We evaluated all consecutive CDH survivors born between January 2009 and December 2017 participating in our prospective long-term follow-up program with a standardized protocol.

**Results:**

A total of 337 patients were included, with a median follow-up of 4 years. SBO with various underlying causes was observed in 38 patients (11.3%) and significantly more often after open surgery (OS). The majority of SBOs required surgical intervention (92%). Adhesive SBO (ASBO) was detected as the leading cause in 17 of 28 patients, in whom surgical reports were available. Duration of chest tube insertion [odds ratio (OR) 1.22; 95% CI 1.01–1.46, *p* = 0.04] was identified as an independent predictor for ASBO in multivariate analysis. Beyond the cut-off value of 16 days, the incidence of serous effusion and chylothorax was higher in patients with ASBO (ASBO/non-SBO: 2/10 vs. 3/139 serous effusion, *p* = 0.04; 2/10 vs. 13/139 chylothorax, *p* = 0.27). Type of diaphragmatic reconstruction, abdominal wall closure, or ECMO treatment showed no significant association with ASBO. A protective effect of one or more re-operations has been detected (RR 0.16; 95% CI 0.02–1.17; *p* = 0.049).

**Conclusion:**

Thoracoscopic CDH repair significantly lowers the risk of SBO; however, not every patient is suitable for this approach. GoreTex®-patches do not seem to affect the development of ASBO, while median laparotomy might be more favorable than a subcostal incision. Neonates produce more proinflammatory cytokines and have a reduced anti-inflammatory capacity, which may contribute to the higher incidence of ASBO in patients with a longer duration of chest tube insertion, serous effusion, chylothorax, and to the protective effect of re-operations. In the future, novel therapeutic strategies based on a better understanding of the biochemical and cellular processes involved in the pathophysiology of adhesion formation might contribute to a reduction of peritoneal adhesions and their associated morbidity and mortality.

## Introduction

Congenital diaphragmatic hernia (CDH) is a rare malformation of an incompletely formed diaphragm. Depending on the size of the defect and its association with major cardiac anomalies, survival rates vary from 99 to 39% (1). It is assumed that due to advances in treatment and with the application of standardized treatment protocols, the overall survival improved, even in severely diseased infants (2, 3). Therefore, CDH-associated morbidity due to pulmonary hypoplasia, pulmonary hypertension, gastroesophageal reflux, musculoskeletal abnormalities, and impaired neurodevelopment have drawn more attention (2, 4).

Among these long-term sequelae, adhesive small bowel obstruction (ASBO) occurs as a life-threatening event after surgical reconstruction of the diaphragm, but objective data are scarce. In general, the type of surgery and the extent of peritoneal damage are considered the most important risk factors for SBO due to adhesions (5). Also, other triggers for adhesion-related readmissions including peritonitis, previous surgery, or patient age have been described (6). Taken together, reduced fibrinolysis, increased fibrin formation, procoagulatory status, and enhanced inflammation seem to be the most important factors in the pathophysiology of adhesion formation in general (7). In neonates, there are additional factors contributing to the formation of adhesions: a reduced production of anti-inflammatory cytokines and a diminished response to anti-inflammatory stimuli in preterm and post-term infants have been reported (8). Additionally, in neonates with CDH complicated by pulmonary hypertension, increased levels of adhesion molecules that play an important role in the inflammatory and immunologic response were detected (9). Therefore, immaturity of the immune system in the neonatal period and pulmonary hypertension inherent to CDH may support the formation of abdominal adhesions in neonates with CDH due to an imbalance of the humoral and cellular system with proinflammatory tendency.

In adults, postoperative adhesions were found in 93% of patients, who had one or more previous abdominal operations (10). Adhesions can be defined as strands or membranes of fibrous tissue that connect various intra-abdominal organs, which are normally separated (5, 11). However, adhesions can be asymptomatic or cause symptoms, such as abdominal pain, altered bowel habits, bloating, or intestinal obstruction, which may be either partial or complete (12, 13). The ASBO seems to be associated with a substantial risk of morbidity (circulatory disturbances, gangrenous bowel, perforation, need for bowel resection, and septicaemia) (14–18) and mortality in children (14), which is nowadays mainly attributed to overwhelming sepsis or other comorbidities (16).

For children, the reported incidence of postoperative bowel obstruction requiring further laparotomy varies from 3.3 to 8.3% in patients, who had previously undergone laparotomy in the neonatal period (19, 20). In infants undergoing Ladd's procedure for malrotation, which was associated with CDH, duodenal atresia, gastroschisis, or esophageal atresia, ASBO even occurred in 14.9% (20). However, there is a large variation in the incidence of ASBO depending on the procedure, localization within the abdominal cavity, and patient age (21). A higher incidence of up to 4.7% was shown in children younger than 1 year compared to 2.1% for older children (22). The risk of developing ASBO seems even higher in neonates (3.3%), compared to infants (1.9%) or older children (1.7%), but with no statistical significance (19). However, different studies agree that most of the adhesive obstructions developed within 1 year of the previous procedure but were also observed later (20–22).

Regarding patients with CDH, Yokota et al. reported that neonates who underwent subcostal laparotomy for the reconstruction of the diaphragm required re-operation for intestinal adhesion obstruction significantly more often than patients who underwent other neonatal laparotomies (17.6% vs. 6.7%, *p* = 0.02) (23). A previously performed retrospective study on long-term surgical morbidity in CDH survivors presented an incidence of about 20% for SBO, with a mean follow-up of 7.3 years (24).

Especially in CDH, there are other causes for SBO besides adhesive formations, like duodenal kinking, Ladd's bands, volvulus, or incarceration due to recurrence. At least 45% of patients with CDH have an associated intestinal rotation abnormality (25). This abnormal rotation of the embryological midgut leads to a nonfixation of the right colon, resulting in aberrant attempts of fixation (Ladd's bands) and could cause intestinal passage disruption with the clinical presentation of SBO (25, 26). Also, the sole attachment of the intestine predisposes for volvulus (26). Due to these anatomical characteristics inherent to CDH, patients show a higher risk for volvulus. In 0.3% of CDH survivors, a volvulus occurred within 1.5 years after the reconstruction of the diaphragm (27).

This longitudinal cohort study aimed at identifying the incidence of SBO and ASBO as well as risk factors of surgical, pre-, and postoperative treatment in children with neonatal repair of CDH.

## Materials and Methods

### Study Group

Consecutive neonates with CDH born from 1 January 2009 to 31 December 2017 and treated at our neonatal intensive care unit (NICU) at the Department of Neonatology of the University Children's Hospital Mannheim, University of Heidelberg, were included in this prospective follow-up study. This study was approved by our local ethics committee (2018-592N-MA) and informed consent was obtained from parents. Our standardized long-term follow-up program has been designed to observe the development of CDH survivors from their childhood until adolescence (Table 1).

**Table 1:** Schedule of our standardized follow-up program.

	**Birth**	**$\frac{1}{2}$ y**	**1 y**	**2 y**	**4 y**	**6 y**	**10 y**	**14 y**	**18 y**
Chest X-ray	X	X	X	-	X	X	-	X	-
ECG	X	X	X	X	X	X	X	X	X
Cardiac ECHO	X	X	X	X	X	X	X	X	X
MRI	-	-	-	ECMO	-	-	X	-	-
Lowdose CT	-	-	-	Non ECMO	-	-	-	-	X
Pulmonary function	-	-	-	-	-	X	X	X	X
Ophthalmology	X	-	X	-	-	-	-	-	-
Hearing test	X	-	X	-	-	-	-	-	-
Neurodevelopmental assessment	-	X	X	X	X	X	-	-	-

*y, year; ECG, electrocardiogram; ECHO, echocardiography; MRI, magnetic resonance imaging; CT, computed tomography; ECMO, extracorporeal membrane oxygenation*.

Treatment of all infants born with CDH was based on the guidelines of the CDH-Euro-Consortium (28, 29). Surgical repair of all patients has been performed after hemodynamic stabilization. The approach (midline laparotomy vs. minimal-invasive) was chosen depending on the estimated size of the defect and cardiopulmonary stability of the patient. In patients undergoing laparotomy, a cone-shaped GoreTex®-patch was used for larger defects to create a tension-free closure of the diaphragm (30). Also, if the primary closure of the laparotomy would be too tight, a GoreTex®-patch was implanted into the abdominal wall to prevent abdominal compartment syndrome. Also, for patients with a minimal-invasive reconstruction of the diaphragm, GoreTex®-patches were used in cases with a missing lateral diaphragmatic rim.

Patients, who underwent diaphragmatic reconstruction at another institution or received surgical treatment after 28 days of life, were excluded. Since SBO has been reported to develop mainly within 1 year after the previous surgery, another exclusion criterion was the follow-up of <1 year in patients without SBO. Data were collected until October 2019 and analyzed for SBO and possible risk factors of demographics, surgical, and pre- and post-operative treatment (ethics vote 2019-1151R).

Adhesive SBO has been defined as a partial or complete intestinal obstruction depending on the symptomatology and eligibility for conservative treatment to achieve relief of symptoms and re-establishment of enteral nutrition. Conservative treatment comprised abstinence from oral food, placement of a nasogastric tube, repeated enemas, and parenteral rehydration under close clinical re-evaluation. In cases with signs of impaired circulation or suspected perforation, deterioration of symptoms and/or missing improvement under conservative treatment over more than 3 days or in patients with suspected volvulus or CDH-recurrence, the indication for re-operation was made.

### Statistical Analysis

All data were entered into a Microsoft Excel database and patients were pseudonymized by numbers. Quantitative values were presented by median, minimum, and maximum as well as qualitative values by number (*n*) and percentage (%). Therefore, the study cohort was separated into patients with and patients without SBO and ASBO. Differences in the results of these groups were assessed for statistical significance using χ^2^- and Fisher's exact test for qualitative data, or rather U- and *t*-test for quantitative data. A *p* < 0.05 was considered statistically significant. Odds ratio (OR), as a measure of the effect of the characteristics on SBO, and the likelihood were calculated for quantitative data using logistic regression analysis in case of a significant result. For qualitative data, the relative risk (RR) for the occurrence of SBO was described. In addition, we performed a multivariate analysis to demonstrate the independence of possible risk factors. The analysis was performed using SAS v14.2 [Statistical Analysis System, Version 14.2 (SAS Institute Inc., Cary, North Carolina, USA)] with grateful support from the Department of Medical Statistics and Biomathematics, Medical Faculty Mannheim.

## Results

### Study Cohort

**Figure 1:**
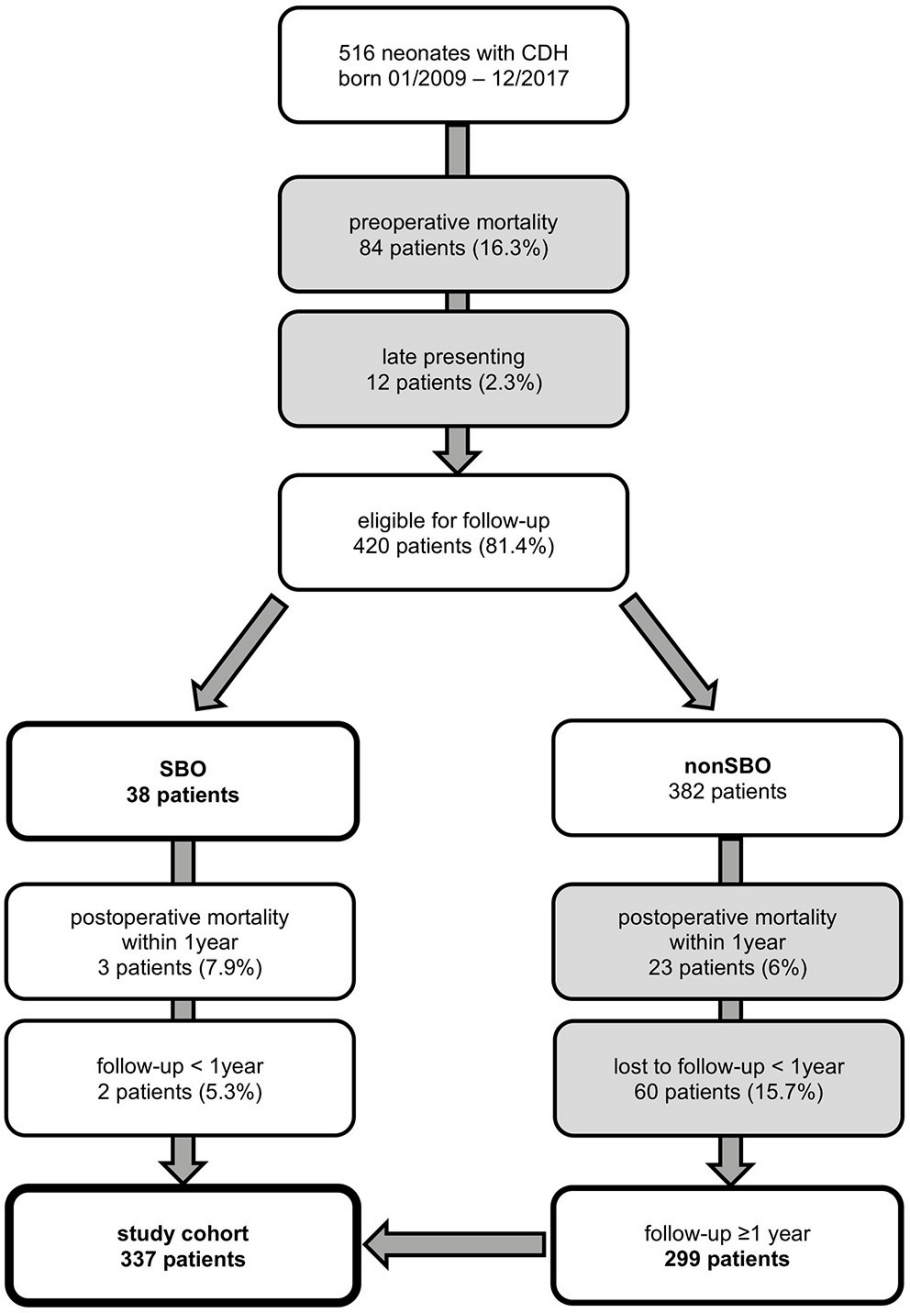
Neonates with congenital diaphragmatic hernia (CDH) born from January 2009 to December 2017 and treated at our institution and participation at follow-up until October 2019 with excluded patients in gray boxes [SBO = small bowel obstruction].

A consort diagram of our study cohort is presented in Figure 1. A total of 516 patients were identified, of which 84 (16.3%) deceased before surgical repair and 12 (2.3%) were late presenting. Therefore, 420 patients were eligible for this study, of whom 26 deceased within the first year of life. Besides 38 patients who developed SBO, 299 patients without SBO completed at least 1 year of follow-up. Thus, 337 neonates were included for further analysis.

Almost every infant received an antibiotic treatment postnatally (96.1%, *n* = 323). The use of extracorporeal membrane oxygenation (ECMO) was necessary in 139 cases (41.3%) with a median duration of 9.0 days.

### Surgical Treatment and Intraoperative Findings

For detailed information on surgical treatment and intraoperative findings, refer to Table 2. Minimally invasive surgery (MIS) was successfully completed in 20.5% of patients, of whom the majority received a primary thoracoscopy (98.6%). A conventional open approach with a midline laparotomy was performed in 79.5%, of which 21 patients were converted from an initial minimal invasive approach. There was a predominance of left-sided CDH (84.3%), CDH without a hernial sac (86.7%), and posterior localization of the diaphragmatic defect (Bochdalek's foramen, 92.8%). Defect size was intraoperatively classified according to the CDH study group (1) in all neonates and mainly large defects were detected in our cohort (C and D: 56.7%). Accordingly, surgical repair of the diaphragm was performed with a GoreTex®-patch in 80.7% of all patients, whereas primary closure was achieved in 19.3%. The difference regarding the type of CDH repair between patients with open surgery (OS) and MIS was significant (patch: 93.7% OS vs. 30.4% MIS; *p* < 0.0001). To prevent abdominal compartment syndrome, a GoreTex®-patch was implanted in the abdominal wall in 25.4% with midline laparotomy. Intraoperative adhesion prevention was used in 3.4% in OS. Mostly, the initial surgery was performed without contamination (93.2%) as classified by the Centers for Disease Control and Prevention (CDC) (31). A total of 32.1% of patients received additional procedures during the surgical reconstruction of the diaphragm.

**Table 2:** Surgical characteristics and intraoperative findings of the study cohort.

	**Study cohort** ***n =* 337**
Timing of reconstruction in days – median (min.-max.)	6 (0 - 21)^a^
Surgical time in minutes – median (min.-max.)	174 (58 - 388)^b^
Operation at neonatal intensive care unit	202 (59.9)
Surgical approach – n (%)	
Minimally invasive Open	69 (20.5) 268 (79.5)
Side of defect – n (%)	
Left side Right side Bilateral	284 (84.3) 51 (15.1) 2 (0.6)
Liver-up in left sided CDH– n (%)	160 (56.3)
Hernia type – n (%)	
Discontinuity/without hernia sac With hernia sac	292 (86.7) 45 (13.4)
Defect size^c^ – n (%)	
A B C D	31 (9.2) 115 (34.1) 158 (46.9) 33 (9.8)
Anatomic localisation of the defect – n (%)	
Bochdalek Morgagni or Larrey	311 (92.8)^d^ 24 (7.2)
Reconstruction of the diaphragm – n (%)	
Primary closure Patch correction	65 (19.3) 272 (80.7)
Abdominal wall closure with patch – n (%)	68 (20.2)
Intraoperative adhesion prevention – n (%)	
Seprafilm® Fibrin	7 (2.1) 2 (0.6)
Contamination class^e^ – n (%)	
1 2 3	314 (93.2) 22 (6.5) 1 (0.3)
Cases with additional operative procedures – n (%)^f^	108 (32.1)^e^
- Release of duodenal kinking - Resection of Meckel's diverticulum - Resection of accessory spleen - Primary fundopexy - Resection of Ladd's bands - Resection of lung sequestration - Resection of accessory liver tissue - Miscellaneous additional procedures^g^ - Insertion of stoma	38 (11.3) 18 (5.3) 18 (5.3) 16 (4.8) 13 (3.9) 13 (3.9) 12 (3.6) 10 (3.0) 2 (0.6)

*CDH, congenital diaphragmatic hernia*.

*^a^1 data missing*.

*^b^3 data missing*.

*^c^classified by Lally et al. (1)*.

*^d^2 data missing*.

*^e^classified by the CDC: Surgical Wound Classification Grades I–IV (31)*.

*^f^cases received more than one additional procedure*.

*^g^suture of intestinal perforation, resection of intestine, mesentery adaption, closure of tracheoesophageal fistula, lymph node resection, and suture of pericardial defect*.

### Re-operations During Follow-Up

During follow-up, 91 patients (27.0%) underwent secondary surgical procedures in the abdominal or thoracic cavity other than for SBO. In total, 62 CDH survivors (18.4%) had one, 19 (5.6%) had two, and eight (2.4%) had three re-operations. In two complicated cases, one patient received seven and one child received eight re-operations over the years. The median time to the first re-operation was 156 days (range 1-1,972), 402 days (range 39–3,193) to the second, and 691.5 days (185–1,942) to the third. Re-operation due to recurrence occurred in 10.4% (*n* = 35). During follow-up, the implanted abdominal wall patch was excised in 56 of 68 cases (82.4%) and reduced in size in 7 patients (10.3%). For treatment of gastroesophageal reflux, hiatoplasty and fundoplication were performed in 23 cases (6.8%), and other intestinal procedures like resection of the intestine or Meckel's diverticulum, insertion of a jejunal feeding tube or stoma, or pyloromyotomy in 28 cases (8.3%) were performed. A total of 16 patients (17.6%) underwent miscellaneous procedures (reconstruction of umbilical or incisional abdominal wall hernias, resection of tumorous formations, funicolysis of intraabdominal testes, cholecystostomy, laparostomy formation, and partial lung resection due to CPAM or implantation of ventriculoperitoneal shunts). Intraoperative adhesion prevention barriers were used in 17.6% of patients.

### Small Bowel Obstruction

**Figure 2:**
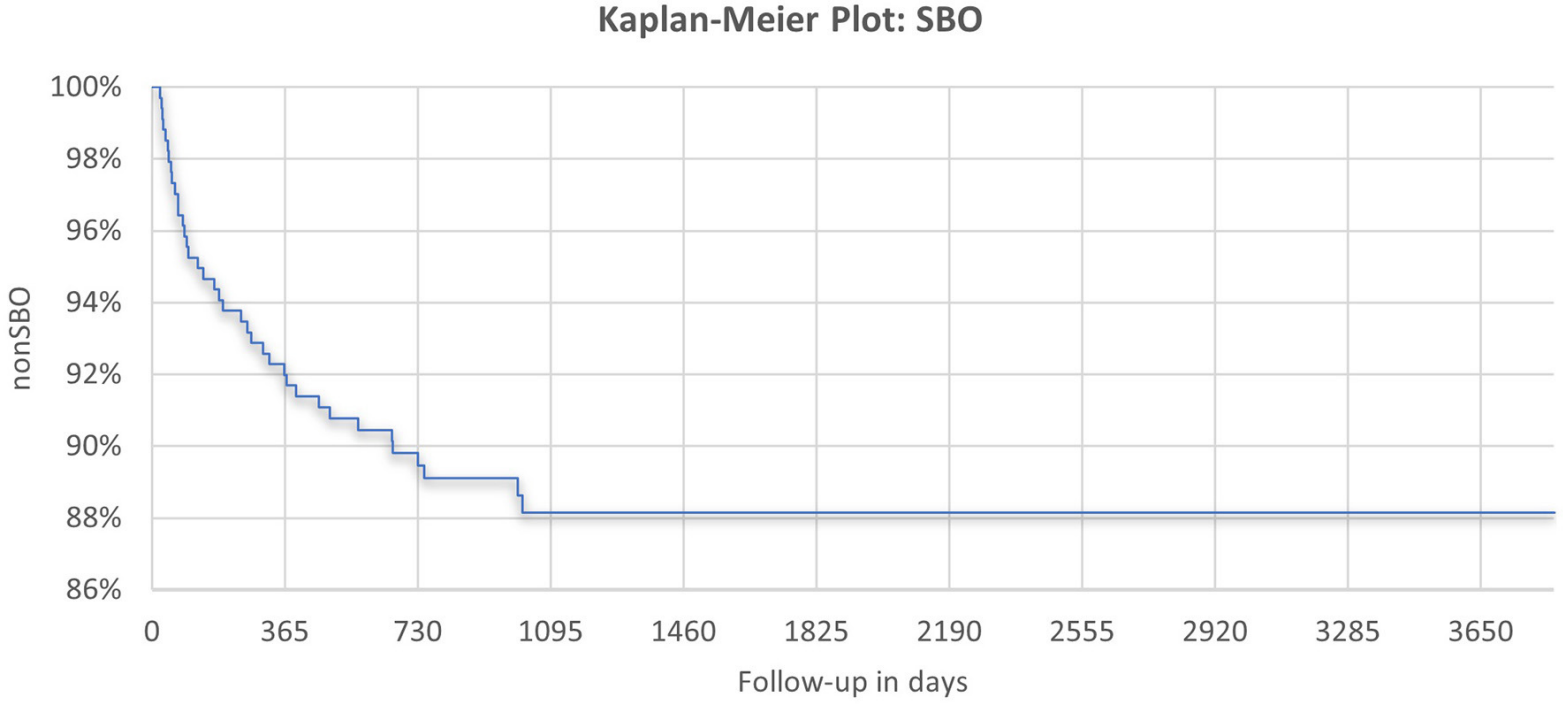
Kaplan–Meier curve for the occurrence of SBO during follow-up with a maximum of 10 years.

During the observation period from January 2009 to October 2019, SBO was observed in a total of 38 patients (11.3%), with a median time to the presentation of 178 days (range, 23–1018). Most were diagnosed in the first year of life (*n* = 27; 71.1%), another seven (18.4%) within the second, and four (10.5%) within the third year after the initial intervention (Figure 2). A total of 10 of 38 children showed a partial obstruction (26.3%), of which three could be treated conservatively (30%). There was one child with recurrent SBO, but with incomplete obstruction and conservative treatment, respectively. In total, 26 children were treated at our institution, and the remaining 12 were treated at an outside hospital. The majority of children presenting with SBO needed surgical treatment (*n* = 35; 92.1%). In seven cases (20%), surgical reports did not reveal a distinct cause, or data are missing due to surgery being performed at an outside hospital. Among those with available surgical reports, adhesive bands were identified as a leading cause (*n* = 17; 60.7%). Furthermore, one or more of the following underlying conditions for the symptoms of SBO could be detected: volvulus (*n* = 5; 17.9%), intestinal kinking (*n* = 5, 17.9%), incarceration due to CDH recurrence (*n* = 3; 10.7%), inner herniation (*n* = 3; 10.7%), and Ladd's bands (*n* = 1; 3.6%).

In 13 patients (46.4%), one or more additional procedures were performed: segmental resection of the intestine (*n* = 7; 25%), antireflux surgery (*n* = 4; 14.3%), jejunal feeding tube placement (*n* = 4; 14.3%), insertion of a stoma (*n* = 2; 7.1%), or laparostomy formation (*n* = 1; 3.6%). Barriers for adhesion prevention were used five times at re-laparotomy (17.9%).

Median follow-up was 3.7 years (range, 0.4–6.7) for the SBO group, 4.2 years (range, 0.4–6.3) for the ASBO group, and 4.1 years (range, 1.0–10.6) for the non-SBO group. We found no significant differences in demographics, patient characteristics, or preoperative treatment between patients with and without SBO.

### Mortality and Morbidity

There was no known mortality associated with the occurrence of SBO or ASBO in our cohort. Bowel resection was necessary in three of 17 patients with ASBO (17.6%).

### Surgical Characteristics, Intraoperative Findings, and Postoperative Treatment in SBO

Details of surgical characteristics and intraoperative findings are displayed in Table 3. The only significant difference between patients with SBO and non-SBO was detected for the surgical approach: a higher incidence of SBO was observed after open surgery (OS 13.1% vs. MIS 4.4%; *p* = 0.04). The type of diaphragmatic reconstruction (primary vs. patch-repair) showed no difference, neither in the total cohort nor in patients after median laparotomy (4/17 (23.5%) primary vs. 31 of 251 (12.4%) patch-repair, *p* = 0.25). The difference in SBO between patients with and without implantation of an abdominal wall patch (AWP) after laparotomy did not reach significance [4/68 patients with AWP (5.9%) vs. 31/200 patients without AWP (15.5%), *p* = 0.06].

**Table 3:** Differences in surgical characteristics and intraoperative findings between patients with SBO and non-SBO.

	**SBO** ***n =* 38**	**Non-SBO** ***n =* 299**	***p*-value**
Timing of reconstruction in days	4.5 (0–18)	6 (0–21)^a^	0.42
Median (min.-max.)			
Surgical time in minutes – median (min.-max.)	173 (95-283)^b^	174 (58–388)^c^	0.82
Operation at neonatal intensive care unit	24 (63.2)	178 (59.5)	0.67
Surgical approach – n (%)			
Minimally invasive Open	3 (7.9) 35 (92.1)	66 (22.1) 233 (77.9)	**0.04**
Side of defect – n (%)			
Left side Right side Bilateral Liver-up in left sided CDH– n (%)	36 (94.7) 2 (5.3) 0 (0.0) 22 (61.1)	284 (95) 49 (16.4) 2 (0.7) 138 (48.6)	0.15 0.54
Hernia type – n (%)			
Discontinuity/without hernia sac with hernia sac	32 (84.2) 6 (15.8)	260 (87) 39 (13)	0.64
Defect size^d^ – n (%)			
A B C D	5 (13.1) 11 (28.9) 21 (55.3) 1 (2.6)	26 (8.7) 104 (34.8) 137 (45.8) 32 (10.7)	0.25
Anatomic localisation of the defect – n (%)			
Bochdalek Morgagni or Larrey	35 (94.6)^e^ 2 (5.3)	276 (92.6)^f^ 22 (7.4)	1.00
Reconstruction of the diaphragm – n (%)			
Primary closure Patch correction	6 (15.8) 32 (84.2)	59 (19.7) 240 (80.3)	0.56
Abdominal wall closure with patch – n (%)	4 (10.5)	64 (21.4)	0.12
Intraoperative adhesion prevention– n (%)			
Seprafilm® Fibrin	0 (0.0) 0 (0.0)	7 (2.3) 2 (0.7)	1.00 1.00
Contamination class^g^ – n (%)			
1 2 3	35 (94.6) 3 (5.4) 0 (0.0)	279 (93.3) 19 (6.4) 1 (0.3)	0.77
Cases with additional operative procedures – n (%)^h^	13 (34.2)	95 (31.8)	0.76
- Release of duodenal kinking - Resection of meckel's diverticulum - Resection of accessory spleen - Hiatoplasty and fundoplication for GER - Resection of ladd's bands - Resection of lung sequestration - Resection of accessory liver - Miscellaneous additional procedures^i^ - Insertion of stoma	4 (10.5) 2 (5.3) 3 (7.9) 3 (7.9) 1 (2.6) 2 (5.3) 0 (0.0) 2 (5.3) 0 (0.0)	34 (11.4) 16 (5.4) 15 (5.0) 13 (4.3) 12 (4.0) 11 (3.7) 12 (4.0) 8 (2.7) 2 (0.7)	1.00 1.00 0.44 0.41 1.00 0.65 0.37 0.31 1.00

*SBO, small bowel obstruction; non-SBO, no small bowel obstruction; CDH, congenital diaphragmatic hernia; GER, gastroesophageal reflux*.

*^a^1 missing data*.

*^b^1 missing data*.

*^c^2 missing data*.

*^d^classified by lally et al. (1)*.

*^e^1 missing data*.

*^f^1 missing data*.

*^g^classified by the cdc: surgical wound classification grades i–iv (31)*.

*^h^cases received more than one additional procedure*.

*^i^suture of intestinal perforation, resection of intestine, mesentery adaption, closure of tracheoesophageal fistula, lymph node resection, and suture of pericardial defect. Bold values are significant*.

Details regarding postoperative treatment are summarized in Table 4. Significant differences between patients with SBO and non-SBO were found for time to full enteral feeding (*p* = 0.02) and the duration of the chest tube insertion (*p* = 0.02). We identified a significant correlation between the duration of the chest tube insertion and the corresponding findings (*p* < 0.0001), whereby chylothorax showed the longest duration with a median of 10 days. Also, the duration of the chest tube insertion among chylothorax was longer in patients with SBO than in patients with non-SBO (median 17 days vs. 10 days; *p* = 0.23). Chylothorax was found to be predominant (17/24, 70.8%) when focusing on the findings beyond the cut-off value of 16 days. In addition, 4 of 17 (23.5%) patients with SBO compared to 13 of 139 (9.4%) patients without SBO had a chest tube for chylothorax beyond 16 days, but this correlation did not reach statistical significance (*p* = 0.09).

**Table 4:** Differences in postoperative treatment and concerning re-operations between SBO- and non-SBO-patients.

	**SBO** ***n* = 38**	**Non-SBO** ***n* = 299**	**p*-*value**
Duration of invasive ventilation in days	22.5 (6–157)	21.0 (1–143)^a^	0.71
Median (min.-max.)			
Duration of NIV in days – median (min.-max.)	10.5 (0-147)	7 (0-176)^b^	0.40
Postoperative nutrition – n (%)			
Breast milk Formula	18 (47.4) 6 (15.8)	136 (46.1)^c^ 60 (20.3)	0.79
Breast milk and formula	14 (36.8)	99 (33.6)	
Timing of postoperative oral nutrition in days	4 (1–12)	4 (1–49)^d^	0.62
– Median (min.-max.)			
Time to full enteral feeding in days	30 (9–99)^e^	23 (3–194)^f^	**0.02**
– Median (min.-max.)			
Insertion of chest tube – n (%)			
Intraoperative/preventive Secondary	1 (2.6) 16 (42.1)	10 (3.3) 130 (43.5)	0.95
Findings of chest tube – n (%)			
Serous effusion Chylothorax Pneumothorax No findings Empyema	9 (53.0) 7 (41.2) 1 (5.9) 0 (0.0) 0 (0.0)	65 (46.8) 56 (40.3) 14 (10.1) 3 (2.2)^7^ 1 (0.7)	1.00
Duration of chest tube insertion in days	11 (4–39)	7 (1–39)^h^	**0.02**
– Median (min.–max.)			
Duration of antibiotic therapy in days	33 (7-114)^i^	30.0 (4-174)^j^	0.71
– Median (min.-max.)			
Escalation of antibiotic therapy – n (%) time to discharge in days – median (min.-max.)	24 (63.2) 61.5 (7–210)	169 (57.3)^k^ 53 (1–270)^l^	0.49 0.32
Number of re-operations – n (%)			
0 1 2 3 7 8 ≥1	36 (94.7) 1 (2.6) 1 (2.6) 0 (0.0) 0 (0.0) 0 (0.0) 2 (5.3)	210 (70.2) 61 (20.4) 18 (6.0) 8 (2.7) 1 (0.3) 1 (0.3) 89 (29.8)	**0.03** **0.001**
timing to 1st re-operation in days	281 (1–561)	156 (12–1972)	0.61
– median (min.-max.)			
timing to 2nd re-operation in days	392 (-)	468.5 (39-3193)	0.90
— median (min.-max.)			
timing to 3rd re-operation in days	-	691.5 (185-1942)	-
— median (min.-max.)			

*SBO, small bowel obstruction; non-SBO, no small bowel obstruction; NIV, non-invasive ventilation. Bold values are significant*.

*^a^2 data missing*.

*^b^4 data missing*.

*^c^4 data missing*.

*^d^7 data missing*.

*^e^5 data missing*.

*^f^18 data missing*.

*^g^1 data missing*.

*^h^1 data missing*.

*^i^1 data missing*.

*^j^6 data missing*.

*^k^4 data missing*.

*^l^1 data missing*.

Concerning re-operations during the observation period, SBO occurred in two of 91 patients (2.2%), who underwent one or more surgical procedures other than for SBO. In contrast, 36 of 246 patients (14.6%) without re-operation developed SBO (*p* = 0.001). No effect of timing on the re-operation could be identified (Table 4).

### Logistic Regression and Multivariate Analysis

The statistically significant effects of the surgical approach, the duration of chest tube insertion, and re-operation on SBO could be confirmed by further analysis and proved to be independent predictors (increase duration of chest tube insertion by 3 days: OR 1.33; 95% CI 1.10–1.60; *p* = 0.003; no re-operation: OR 19.9; 95% CI 2.28–174.24; *p* = 0.01). Midline laparotomy in comparison to thoracoscopy significantly increased the relative risk of SBO 3-fold (RR 3.00, 95% CI 0.95–9.48; *p* = 0.04). In contrast, performing one or more re-operations had a protective effect and reduced the risk of SBO by 85% (RR 0.15; 95% CI 0.04–0.61; *p* = 0.001). The likelihood of SBO was 14.6% in patients without re-operation, decreased to 1.6% with one re-operation, and increased slightly to 5.3% with two re-operations.

In the attempt to identify risk factors for the formation of adhesions, further analysis of 17 ASBO patients in comparison to 299 patients with non-SBO was performed.

### Adhesive Small Bowel Obstruction

Focusing on adhesion formation, which caused 17 of 28 SBOs with available surgical reports (60.7%), we found significant predictors for ASBO. No significant difference concerning demographics, patient characteristics, and preoperative treatment could be detected (Table 5). Regarding surgical characteristics and intraoperative findings (Table 6), all 17 patients with ASBO received a midline laparotomy, whereas no child with thoracoscopy developed ASBO. Therefore, we determined that midline laparotomy significantly increased the risk of developing ASBO by 28% (95% CI 1.20–1.36; *p* = 0.03). Defect size also showed a significant difference with predominantly larger defect sizes in patients with ASBO. In logistic regression analysis, this did not show an effect. With regard to postoperative treatment, the time to full enteral feeding was significantly longer in patients with ASBO but could not be confirmed by logistic regression analysis (Table 7). In accordance with our previous findings concerning SBO in general, there was a correlation between the duration of the chest tube insertion for serous effusion and chylothorax with a longer duration in our ASBO cohort as compared to patients with non-SBO. After a cut-off value of 16 days, this difference was significant for serous effusion (*p* = 0.04), but not for chylothorax (*p* = 0.31). Due to the small number of patients, no further statistical analysis could be performed (Table 8). A significantly lower incidence of ASBO was observed in patients, who underwent secondary surgeries during follow-up, with a trend concerning excision of the abdominal wall patch (Table 9). The duration of the chest tube insertion also significantly increased the risk for ASBO (increase duration by 3 days: OR 1.22; 95% CI 1.01–1.46; *p* = 0.04), while re-operations were associated with a significantly decreased risk of ASBO (one or more re-operations: RR 0.16; 95% CI 0.02–1.17; *p* = 0.049) (Table 10). Both factors were found to be independent predictors in multivariate analysis (no re-operation: OR 10.05; 95% CI 1.13–89.30; *p* = 0.04) (Table 11). Also, with the increasing duration of chest tube insertion, the probability of ASBO was higher in patients without re-operation than in patients with one or more re-operations (Table 12).

**Table 5:** Differences in demographics, patient characteristics, and preoperative treatment between patients with ASBO- and non-SBO.

	**ASBO** ***n* = 17**	**Non-SBO** ***n* = 299**	***p*-value**
Follow-up in years — median (min.-max.)	4.2 (0.4–6.3)	4.1 (1.0–10.6)	0.86
Sex – n (%)			
Male Female	5 (29.4) 12 (70.6)	127 (42.5) 172 (57.5)	0.29
Birth mode – n (%)			
Vaginal Caesarean section	3 (17.7) 14 (82.3)	68 (23.6)^a^ 220 (76.4)	0.77
Date of delivery in gw	37+6	38+0	0.82
– Median (min.-max.)	(35+0 – 41+5) ^b^	(27+3 – 41+4)^c^	
Amnion infection syndrome – n (%)	0 (0.0)	6 (2.0)^d^	1.00
Birth weight in kg – median (min.-max.)	2.9 (1.9-3.9)^e^	3.0 (1.4-4.6)^f^	0.94
Birth hight in cm – median (min.-max.)	49.0 (40.0-55.5)^g^	50.0 (40.0-63.0)^h^	0.41
Outborn – n (%)	2 (11.8)	48 (16.1)	1.00
Associated structural malformations or syndromes – n (%)^i^	10 (58.8)	140 (46.8)	0.34
Urinary and genital Minor cardiovascular Syndromes Musculoskeletal Malformation of the kidneys Omphalocele/abdominal hernia Major cardiovascular Cerebral Hepatobiliary Esophageal atresia/-stenosis Trachea-/bronchomalacia	5 (29.4) 2 (11.8) 2 (11.8) 1 (5.9) 1 (5.9) 1 (5.9) 0 (0.0) 0 (0.0) 0 (0.0) 0 (0.0) 0 (0.0)	47 (15.7) 37 (12.4) 14 (4.7) 21 (7.0) 20 (6.7) 3 (1.0) 16 (5.4) 13 (4.4) 7 (2.3) 2 (0.7) 3 (1.0)	0.17 1.00 0.21 1.00 1.00 0.20 1.00 1.00 1.00 1.00 1.00
Antibiotics since delivery – n (%)	17 (100.0)	286 (96.0)^j^	1.00
Use of FETO – n (%)	2 (11.8)	22 (7.5)^k^	0.63
Use of ECMO – n (%)	9 (52.9)	121 (40.5)	0.31
Duration of ECMO in days	12 (6–14)	9 (4-22)	0.22
– Median (min.-max.)			

*ASBO, adhesive small bowel obstruction; non-SBO, no small bowel obstruction; gw, gestational week; FETO, fetal endoscopic tracheal occlusion; ECMO, extracorporeal membrane oxygenation*.

*^a^11 data missing*.

*^b^1 data missing*.

*^c^11 data missing*.

*^d^3 data missing*.

*^e^1 data missing*.

*^f^6 data missing*.

*^g^3 data missing*.

*^h^27 data missing*.

*^i^cases had more than one associated anomaly*.

*^j^1 data missing*.

*^k^5 data missing*.

**Table 6:** Differences in surgical characteristics and intraoperative findings between patients with ASBO and non-SBO.

	**ASBO** ***n* = 17**	**Non-SBO** ***n* = 299**	***p*-value**
Timing of reconstruction in days	4 (2–16)	6 (0–21)^a^	0.77
– Median (min.-max.)			
Surgical time in minutes – median (min.-max.)	181.5 (95–255)^b^	174 (58–388)^c^	0.83
Operation at neonatal intensive care	12 (70.6)	178 (59.5)	0.37
Surgical approach – n (%)			
Minimally invasive Open	0 (0.0) 17 (100.0)	66 (22.1) 233 (77.9)	**0.03**
Side of defect – n (%)			
Left side Right side Bilateral	15 (88.2) 2 (11.8) 0 (0.0)	248 (82.9) 49 (16.4) 2 (0.7)	1.00
Liver-up in left sided CDH– n (%)	9 (60.0)	138 (55.7)	0.74
Hernia type – n (%)			
Discontinuity/without hernia sac With hernia sac	15 (88.2) 2 (11.8)	260 (87) 39 (13)	1.00
Defect size^d^ – n (%)			
A B C D	2 (11.8) 2 (11.8) 13 (76.5) 0 (0.00)	26 (8.7) 104 (34.8) 137 (45.8) 32 (10.7)	**0.04**
Anatomic localisation of the defect – n (%)			
Bochdalek Morgagni or Larrey	16 (100.0)^e^ 0 (0.00)	276 (92.6)^f^ 22 (7.4)	0.61
Reconstruction of the diaphragm – n (%)			
Primary closure Patch correction	2 (11.8) 15 (88.2)	59 (19.7) 240 (80.3)	0.54
Abdominal wall closure with patch in open surgery	3 (17.6)	64 (27.5)	0.57
– n (%)			
Intraoperative adhesion prevention– n (%)			
Seprafilm® fibrin	0 (0.0) 0 (0.0)	7 (2.3) 2 (0.7)	1.00 1.00
Contamination class^g^ – n (%)			
1 2 3	16 (94.1) 1 (5.9) 0 (0.0)	279 (93.3) 19 (6.4) 1 (0.3)	1.00
Cases with additional operative procedures – n (%)^h^	7 (41.2)	95 (31.8)	0.42
- Release of duodenal kinking - Resection of Meckel's diverticulum - Resection of accessory spleen - Fundoplication as GER-prevention - Resection of Ladd's bands - Resection of lung sequestration - Resection of accessory liver - Miscellaneous additional procedures^i^ - Insertion of jejunal feeding tube/stoma	3 (17.7) 0 (0.0) 1 (5.9) 2 (11.8) 1 (5.9) 1 (5.9) 0 (0.0) 1 (5.9) 0 (0.0)	34 (11.4) 16 (5.4) 15 (5.0) 13 (4.3) 12 (4.0) 11 (3.7) 12 (4.0) 8 (2.7) 2 (0.7)	0.43 1.00 0.60 0.19 0.52 0.49 1.00 0.40 1.00

*ASBO, adhesive small bowel obstruction; non-SBO, no small bowel obstruction. Bold values are significant*.

*^a^1 missing data*.

*^b^1 missing data*.

*^c^2 missing data*.

*^d^classified by Lally et al. (1)*.

*^e^1 missing data*.

*^f^1 missing data*.

*^g^classified by the CDC: Surgical Wound Classification Grades I–IV (31)*.

*^h^cases received more than one additional procedure*.

*^i^suture of intestinal perforation, resection of intestine, mesentery adaption, closure of tracheoesophageal fistula, lymph node resection, and suture of pericardial defect*.

**Table 7:** Differences in postoperative treatment between patients with ASBO and non-SBO.

	**ASBO** ***n* = 17**	**Non-SBO** ***n* = 299**	***p*-value**
Duration of invasive ventilation in days	27.0 (7–138)	21.0 (1–143)^a^	0.35
– Median (min.-max.)			
Duration of NIV in days – median (min.-max.)	10.5 (0–147)	7 (0–176)^b^	0.12
Postoperative nutrition – n (%)			
Breast milk Formula	8 (47.1) 3 (17.7)	136 (46.1)^c^ 60 (20.3)	0.96
Breast milk and formula	6 (35.3)	99 (33.6)	
timing of postoperative oral nutrition in days	4 (1–12)	4 (1–49)^d^	0.81
– Median (min.-max.)			
Time to full enteral feeding in days	33 (12–70)^e^	23 (3–194)^f^	**0.02**
– median (min–max.)			
Insertion of chest tube – n (%)			
Intraoperative/preventive	1 (5.9)	10 (3.3)	0.59
Secondary	9 (52.9)	130 (43.5)	
Findings of chest tube – n (%)	10	139^g^	
Serous effusion Chylothorax pneumothorax no findings empyema	6 (60.0) 3 (30.0) 1 (10.0) 0 (0.0) 0 (0.0)	65 (46.8) 56 (40.3) 14 (10.1) 3 (2.2) 1 (0.7)	0.85
duration of chest tube insertion in days	12.5 (4–39)	7 (1-39)^h^	0.13
– median (min.-max.)			
findings of second chest tube – n (%)	0	11	
serous effusion pneumothorax chylothorax empyema	0 (0.0) 0 (0.0) 0 (0.0) -	4 (36.4) 4 (36.4) 3 (27.3) -	1.00 -
duration of antibiotic therapy in days			
– median (min.-max.)	39 (10–114)^i^	30.0 (4–174)^j^	0.30
escalation of antibiotic therapy – n (%)	11 (64.7)	169 (57.3)^k^	0.55
time to discharge in days – median (min–max.)	63 (25–184)	53 (1–270)^l^	0.33

*ASBO, adhesive small bowel obstruction; non=SBO, no small bowel obstruction; NIV, non-invasive ventilation. Bold values are significant*.

*^a^2 data missing*.

*^b^4 data missing*.

*^c^4 data missing*.

*^d^7 data missing*.

*^e^1 data missing*.

*^f^18 data missing*.

*^g^1 data missing*.

*^h^1 data missing*.

*^i^1 data missing*.

*^j^6 data missing*.

*^k^4 data missing*.

*^l^1 data missing*.

**Table 8:** Correlation between findings of pleural effusion and duration of chest tube insertion.

	**ASBO** ***n* = 10**	**Non-SBO** ***n* = 139^**a**^**	***p*-value**
Duration of chest tube insertion in correlation to findings in days – median (min.-max.)			
chylothorax empyema serous effusion pneumothorax	17 (6–20) - 7.5 (4–39) 25 (-)	10 (2–39) 9 (-) 6 (1–25) 5.5 (1–31)	
findings among chest tube with duration > 16 days – n (%)	5 (50.0)	17 (12.3)	**0.01**
chylothorax serous effusion pneumothorax	2 (20.0) 2 (20.0) 1 (10.0)	13 (9.4) 3 (2.2) 1 (0.7)	0.27 **0.04** 0.13

*ASBO, adhesive small bowel obstruction; non-SBO, no small bowel obstruction. Bold values are significant*.

*^a^1 data missing*.

**Table 9:** Re-operations during follow-up concerning patients with ASBO and non-SBO.

	**ASBO** ***n* = 17**	**Non-SBO** ***n* = 299**	***p*-value**
Number of re-operations – n (%)			
0 1 2 3 7 8 ≥1	16 (94.1) 0 (0.0) 1 (5.9) 0 (0.0) 0 (0.0) 0 (0.0) 1 (5.9)	210 (70.2) 61 (20.4) 18 (6.0) 8 (2.7) 1 (0.3) 1 (0.3) 89 (29.8)	0.21 **0.049**
Timing to 1^st^ re-operation in days	1 (-)	156 (12–1972)	0.09
– Median (min.-max.)			
Timing to 2^nd^ re-operation in days	392 (-)	468.5 (39–3193)	0.90
— Median (min.-max.)			
Timing to 3^rd^ re-operation in days	-	691.5 (185–1,942)	-
— Median (min.-max.)			
Procedure			
- excision of abdominal wall patch^a^ – n (%) - change of abdominal wall patch^a^ – n (%) - recurrence – n (%) - other intestinal procedure^b^ – n (%) - hiatoplasty and fundoplication – n (%) intraoperative adhesion prevention – n (%)	1 (33.3) 1 (33.3) 0 (0.0) 0 (0.0) 0 (0.0) 0 (0.0)	55 (85.9) 6 (9.4) 31 (10.4) 28 (9.4) 22 (7.4) 16 (5.4)	0.07 0.29 0.39 0.38 0.62 1.00

*ASBO, adhesive small bowel obstruction; non-SBO, no small bowel obstruction. Bold values are significant*.

*^a^percentage referred to patients with abdominal wall patch (ASBO: n = 3, non-SBO: n = 64)*.

*^b^resection of Meckel's diverticulum, resection of intestine/stoma, insertion of jejunal feeding tube, pyloromyotomy*.

**Table 10:** Predictors of adhesive small bowel obstruction (ASBO) using univariate analysis and logistic regression.

	**RR/OR (95% CI)**	***p*-value**
defect size B vs. A	0.25 (0.03–1.86)	0.18
defect size C vs. A	1.23 (0.26–5.80)	0.79
surgical approach (midline laparotomy)	1.28 (1.20–1.36)^a^	**0.03**
≥1 re-operations	0.16 (0.02–1.17)	**0.049**
excision of abdominal wall patch	0.10 (0.01–0.99)	0.07
time to full enteral feeding in days (+1)	1.01 (1.00–1.03)	0.11
duration of chest tube insertion in days (+3)	1.22 (1.01–1.46)	**0.04**

*ASBO, adhesive small bowel obstruction; RR, relative risk; OR, odds ratio; CI, confidence interval. Bold values are significant*.

*^a^RR could not be calculated because all patients with ASBO underwent midline laparotomy. Therefore, column risk was determined: (17/17) MIS / (233/299) median laparotomy = 1.28*.

**Table 11:** Independent predictors of adhesive small bowel obstruction (ASBO) using multivariate analysis.

	**OR (95% CI)**	***p*-value**
No re-operation vs. ≥1 re-operations	10.05 (1.13–89.30)	**0.04**
duration of chest tube insertion in days (+3)	1.29 (1.06–1.58)	**0.01**

*ASBO, adhesive small bowel obstruction; OR, odds ratio; CI, confidence interval. Bold values are significant*.

**Table 12:** Likelihood of adhesive small bowel obstruction (ASBO) as a function of the duration of chest tube insertion and the number of re-operations.

	**Likelihood in %,n** **no re-operation**	**Likelihood in %,** **≥1 re-operation**
Duration of chest tube insertion in days		
0 2 4 6 8 10 12 14 16 18 20 22 24 26 28 30	4.39 5.17 6.08 7.14 8.37 9.78 11.41 13.26 15.37 17.74 20.38 23.31 26.52 30.00 33.73 37.67	0.46 0.54 0.64 0.76 0.90 1.07 1.27 1.50 1.77 2.10 2.48 2.94 3.47 4.09 4.82 5.67

*ASBO, adhesive small bowel obstruction*.

## Discussion

Our study seems to confirm that SBO represents an important cause of morbidity after neonatal repair of CDH. We determined an incidence of 11.3% during a prospective 10-year observation period of 337 CDH survivors and identified many different underlying causes. Furthermore, independent risk factors for developing ASBO could be identified: patients requiring a midline laparotomy for the reconstruction of CDH showed a higher risk than patients after minimally invasive repair. Also, the duration of the chest tube insertion was independently predictive of ASBO. In contrast, subsequent re-operations revealed an unexpected protective effect.

In general, literature reports focussing on SBO in children are scarce and only a few studies mention SBO as a complication after CDH repair. To make interpretation and comparison even more difficult, there is often no differentiation between SBO with a broad spectrum of possible causative conditions and ASBO. Mainly retrospective studies with small numbers of patients are available. These are difficult to compare due to the lack of standardized follow-up and varying length of follow-up and thus the true incidence of ASBO in patients with CDH is still unknown. Identification of risk factors is impaired for the same reasons. Literature reports an incidence of SBO from 3 to 20% in patients with CDH who have a wide range of follow-up periods (32). However, the incidence of postoperative bowel obstruction in this specific population seems to be considerably higher than in neonates undergoing laparotomy other than for CDH (19, 20).

Symptoms of SBO were caused by a variety of underlying conditions in our cohort as follows: adhesive bands > volvulus / duodenal kinking > inner herniation / CDH-recurrence > Ladd's bands. Similar findings were observed by Jancelewicz et al. in a prospective follow-up study of 99 CDH survivors: adhesions in 54%, recurrence in 39%, and volvulus in 8% (33). Literature states that CDH predisposes to volvulus, which was the second most frequent cause of SBO, and it occurred in 1.4% of all participants in our study. This data correlate with other reports that identified an incidence of 0.3–1.0% (27, 33). Interestingly, Ward et al. found that the prophylactic Ladd procedure, which was assumed to prevent developing volvulus, was associated with a 3-fold increased risk of subsequent volvulus (27). Furthermore, a higher incidence of SBO was found following surgical interventions for malrotation and of the upper gastrointestinal tract (15, 16). Even though Ladd's procedure is considered routine during CDH repair in many centers, it might be questionable after these findings.

### Adhesion Formation

Adhesions are initially a normal step of the repair mechanisms after peritoneal damage, but an imbalance among fibrinolysis, fibrin formation, coagulation, and inflammation results in persistent fibrous bands (7, 34, 35). The pathophysiological processes behind adhesive formations are still the subject of current research. After a peritoneal injury, a fibrinous exudate is formed as the first step. The formation of fibrin is the result of the coagulation cascade, which can be initiated by tissue factors. On the other hand, fibrinolysis activated by plasmin induces fibrin degradation and is enabled by tissue plasminogen activator (tPA) and urokinase-like plasminogen activator (uPA). Through this cascade, the fibrinous formations should be resorbed within days. Tissue factors as well as tPA, uPA. and their inhibitor, plasminogen activator inhibitors group 1 (PAI-1) are expressed by the mesothelial cells of the peritoneum, while inflammatory cytokines, such as interleukin-1 (IL-1) and tumor necrosis factor-alpha (TNF-α), cause an imbalance with a tendency to fibrin deposition (7, 35). If it remains for too long, the fibrin becomes organized into fibrous strands or membranes, consisting of collagen, blood vessels, and nerves (35).

In addition to cytokines and other mediators, the cellular response to injury may also play a role. Recruitment of neutrophils, as the main subgroup of leukocytes, is the first response to trauma. Neutrophils form neutrophil extracellular traps (NETs) and their influence has been described in various pathologies (36, 37). The NETs are also able to modulate the immune response to support inflammatory processes. Therefore, high levels of NETs have been found in the adhesive tissue.

Further influencing factors expose neonates and especially those with pulmonary hypertension to a greater risk of developing adhesions after laparotomy (8, 9). Peritonitis has been described as a possible risk factor for the formation of adhesions (6). Accordingly, in patients with neonatal laparotomy due to inflammatory conditions like necrotizing enterocolitis, a predominance of dense adhesions has been discovered during re-laparotomy for SBO (15).

In our cohort, CDH repair was performed with no contamination in the vast majority of patients so an additive effect of peritoneal inflammation on the formation of adhesions seems less likely. The majority received antibiotic treatment directly after birth. Laboratory parameters of infection or inflammation were not collected in our study, only escalation of antibiotic therapy was evaluated, and it did not differ between patients with SBO and non-SBO. However, this parameter is not a sufficient surrogate parameter for a proinflammatory status. So far, there are no further studies in patients with CDH reporting any of these conditions in correlation with ASBO.

The severity of adhesions was not addressed by our data, due to a lack of a standardized classification system. Coccolini et al. suggested a regimented classification system for adhesions - the peritoneal adhesion index (PAI) - in an effort to standardize their definition and subsequent analysis (38). A survey on its feasibility showed a high acceptance among surgeons (39). In a prospective observational study of postoperative ASBO, Sisodia et al. found that PAI is a sensitive and effective tool for the quantitative assessment of intraabdominal adhesions (40). In addition, PAI has already been used as a variable in several studies (41, 42). Its implementation could provide further information on risk factors and their influence by making it easier to compare the results of different centers.

No improvement concerning the incidence of ASBO after laparotomy in childhood can be observed (16). Besides general surgical principles with minimal and careful handling of the bowel, minimizing the blood loss, and avoiding devascularization and desiccation of the bowel during surgery, other therapeutic strategies based on a better understanding of the underlying pathophysiology of the formation of adhesions should be considered. To achieve the separation of damaged surfaces (35), adhesion barriers, such as the hyaluronic acid-carboxymethylcellulose membrane Seprafilm®, showed a reduced severity of adhesions as well as few abdominal complaints in adults (43). Other adjuncts based on the current research may be introduced in the near future. It has been reported that DNases can dissolve NETs. Accordingly, Heuer et al. observed a significantly reduced formation of NETs in mice treated with DNase1 (44). In addition, DNase Knockout-mice with laparotomy-induced adhesions showed higher levels of NETs and increased adhesion formation, based on the experiments from Boettcher et al., suggesting an important role of DNases in this context. Furthermore, treatment with DNases in Wildtype-mice resulted in a significant reduction in laparotomy-induced adhesions without negatively affecting wound healing. The preprint may be found on Research Square (https://doi.org/10.21203/rs.3.rs-1077792/v1).

In the following section, the risk of ASBO in CDH is critically reviewed in the context of the current literature regarding the potential and proven influencing factors and our findings are discussed accordingly.

### Minimally Invasive Surgery vs. Open Surgery

A significantly increased rate of ASBO after open abdominal reconstruction of the diaphragm was expectedly replicated in our study with an incidence of 6.8% among patients with midline laparotomy and 0% in patients with a minimally invasive approach. The CDH Study Group revealed similar results already for the initial hospital stay. They showed that patients, who underwent MIS, had a five times lower risk of ASBO requiring an operation until discharge than patients with OS (OR 0.19; 95% CI 0.06–0.60, *p* = 0.005). Also in defect size A, a significantly lower risk of ASBO in patients who underwent MIS could be detected, while it was not significant for other defect sizes (45). In contrast to the CDH study group, we did not observe any ASBO after minimal-invasive repair in the long term, but in a smaller cohort.

Thoracoscopy for CDH repair has become the more popular minimally invasive approach than laparoscopy. Only very few studies report on long-term data regarding ASBO in relation to the surgical approach and MIS cohorts are often too small to draw any conclusions (24). Similar to our findings, Jancelewicz (2010) noticed symptoms of SBO only in correlation with CDH recurrence in the MIS cohort and there was no SBO due to adhesions detected (46).

However, not every patient is suitable for minimal-invasive CDH repair. Thoracoscopy poses a greater risk of technical difficulties leading to conversion as well as intraoperative hypercapnia and acidosis, which potentially affects neurological development (47). Also, several studies described significantly higher recurrence rates after thoracoscopic repair of the diaphragm (45, 48). In our minimal invasive cohort, only patients with CDH recurrence (4%) presented with signs of SBO and no ASBO was encountered. Even in patients with thoracoscopic implantation of a diaphragmatic GoreTex® patch (30%), no problems due to ASBO were observed. This might be due to the less-invasive access itself and pleural rather than peritoneal trauma in thoracoscopy. Therefore, in the subset of patients with CDH having smaller defect sizes (A and B), the thoracoscopic approach with meticulous surgical technique to reduce the risk of CDH recurrence may be superior to the open approach with regard to the prevention of adhesive SBO.

### Defect Size

So far, there is only one study reporting on the incidence of SBO in correlation to defect size. Putnam et al. recognized an increasing incidence of of ASBO with larger defect-size during the initial hospital stay, with a significantly higher incidence after open abdominal surgery. In open surgery, the incidence in correlation to defect size was as follows: A, 2.3%; B, 2.7%; C, 6.6%; and D, 7.9% (45). In our longitudinal follow-up, we found the following incidences of ASBO in open surgery: A, 16.7%; B, 2.9%; C, 8.5%; and D, 0%. The subgroups with defect sizes A and D were small. Within the larger subgroups with defect sizes B and C, the difference was not statistically significant (*p* = 0.24). Thus, larger observational cohort studies with longitudinal follow-up will have to be awaited to verify, if defect size by itself or other factors like patch material and surgical access have an impact on the development of adhesive SBO.

### Patch Repair for Diaphragmatic and Abdominal Wall Reconstruction

A metaanalysis of 10 studies and 1,273 patients reported an about twice higher risk of SBO for patients with patch implantation compared to patients with primary closure of the diaphragm (OR 1.90; 95% CI 1.31–2.76). The incidence of SBO was 6.6% after primary repair and 12% after patch repair. There was no differentiation of ABSO or analysis according to different patch materials, but mainly PTFE was used (32). This makes an interpretation difficult since SBO can have multiple different causes as explained above.

In our cohort, the overall incidence of ASBO was only 6.3% in OS with 11.8% after primary repair and 6% after implantation of a diaphragmatic patch. The difference was not significant due to the small number of patients with primary repair in OS. There was also no difference in the high patch rate in patients with ASBO and non-ASBO (ASBO/non-ASBO: 88.2% vs. 94% patch; *p* = 0.3). The high number of patients with large defects in OS (C: 57.1%; D: 12.3%) and therefore requiring a diaphragmatic patch reflects disease-severity being an ECMO center. The majority of patients with a primary repair was operated by minimal-invasive access at our center (73.8%), which is a potential bias. No ASBO was observed in our patients with MIS despite a patch rate of 30%. In contrast to the results of the metaanalysis, the GoreTex® patch by itself might not contribute to a higher risk of ASBO, since we were using the same patch material irrespective of surgical access.

There was also no difference regarding the incidence of ASBO in correlation to the implantation of an abdominal wall patch: about 4.4% in patients with and 7% in patients without an abdominal wall patch (AWP). The rate of an AWP did not differ between both subgroups (AWP: 17.6% ASBO vs. 27.5% non-SBO; *p* = 0.57). To date, there are no further studies explicitly mentioning abdominal wall patches in correlation with ASBO in patients with CDH. It has to be considered that the abdominal wall patch is a surrogate parameter for the severity of CDH. Mostly, severely affected neonates are referred for treatment to ECMO-centers which explains the high rate of AWP implantation in our cohort. Other centers might therefore find a difference concerning this parameter. Nevertheless, we detected a comparatively low incidence of ASBO. Interestingly, a higher incidence of ASBO was shown for neonates with abdominal wall defects (25% in 59 patients with gastroschisis vs. 13% in 111 patients with omphalocele, *p* = 0.06). Only seven patients received a prosthetic mesh in this cohort. In multivariate analysis, sepsis and fascia dehiscence were identified as independent predictors (18). Thus, the implantation of an AWP (GoreTex®) in severely affected neonates with CDH and a hypoplastic abdominal cavity may be more favorable than only skin closure with iatrogenic fascia dehiscence concerning the development of ASBO.

### Patch Material

Even though the lower incidence of ASBO in patients with implantation of a diaphragmatic or abdominal wall GoreTex® patch was not significant, there might still be something about it to be considered. Literature also gives hints to differences in adhesion formation in correlation to patch material. Patients, who received absorbable biosynthethic patches like Surgisis-Gold® (SIS) or AlloDerm® developed a higher rate of SBO than patients with a nonabsorbable mesh like Dacron® or GoreTex® in some studies, but subgroups of patch patients were small (33, 49, 50). Jancelewicz et al. identified SIS as the only significant subtype in univariate logistic regression analysis comparing primary and patch repair (SIS vs. GoreTex® vs. SIS+GoreTex®) with an OR of 8.1 (95% CI 2-28; p = 0.001) (33). On the other hand, no significant difference concerning ASBO between SIS (7%) and GoreTex® (4%) was observed in a retrospective study of 72 patch patients with a minimal follow-up of 30 days (51).

A less adhesive effect of GoreTex® has been reported in 91% of adults, who had undergone laparoscopic ventral incisional hernia repair with GoreTex® mesh implantation. Adhesions were either not present or were filmy and avascular at the timing of re-operation (52).

Furthermore, polytetrafluoroethylene (PTFE), also used for GoreTex®, was studied as a barrier to prevent adhesions in an animal model and was implanted to cover the injured peritoneum after pelvic surgery. The extent of adhesions was significantly less, and fewer animals had adherent intestinal loops compared to a control group, indicating an effective adhesion prevention barrier (53). Therefore, the type of mesh used for the diaphragmatic reconstruction may influence the development of adhesions and consequently ASBO. We hypothesize that the use of GoreTex®-patches may reduce intraabdominal adhesions due to their specific content of expanded PTFE. This would be an added advantage for the use of GoreTex® for diaphragmatic patch repair besides the seemingly lower long-term recurrence rate (54).

### Laparotomy

Furthermore, the way of abdominal access may influence the development of ASBO. Recently, Janssen et al. revealed an incidence of SBO after CDH repair of 20% in 112 patients but did not differentiate for ASBO. Compared to our overall SBO rate of 11.3% in 337 patients, this was significantly higher (*p* = 0.04). The SBO occurred in 19% of 98 patients after subcostal laparotomy and in 21% of 14 patients with either thoracoscopy, thoracotomy, or laparoscopy. The risk of SBO was significantly higher after patch repair in 35 patients (OR 3.5, 95% CI 1.2–10.0) (24). In their study cohort, a higher proportion of open abdominal reconstruction of the diaphragm was performed as compared to our study cohort, but this difference is not statistically significant (87.5% vs. 79.5%; *p* = 0.07). On the other hand, the patch-rate was significantly lower in their cohort (31% vs. 80.7%; *p* < 0.00001). Yokota et al. noticed an intestinal adhesion obstruction of 17.6% in 74 CDH survivors with subcostal laparotomy and 6.7% in 240 patients with other neonatal laparotomies than for CDH (*p* = 0.023) (23). We encountered a lower incidence of 6.3% for ASBO among 268 patients with CDH who underwent midline laparotomy. There is a significant difference between both cohorts: the incidence of ASBO was higher after subcostal laparotomy (*p* < 0.005) despite a lower rate of patch repair (33.8% vs. 93.7%; *p* < 0.00001).

Interestingly, both authors reported a similar patch rate of about 30% and an incidence of (A)SBO of nearly 20% after subcostal laparotomy, whereas a significantly higher patch rate was observed with a significantly lower rate of (A)SBO following the median laparotomy in our cohort. We had a comparable patch rate of 30% in our MIS cohort, which was not associated with ASBO. Since in all these cohorts, solely Goretex® was used as a patch material, this might not affect the rate of ASBO by itself. While it is well-known that postoperative intestinal obstruction is reduced after laparoscopy compared to laparotomy (55), possibly the difference in abdominal access (subcostal vs. midline laparotomy) might also play a role in the development of ASBO that has been neither reported nor investigated so far. An explanation for this finding could be that with subcostal incisions, the abdominal wall muscles have to be divided, whereas these are kept intact using the midline laparotomy. The more invasive abdominal access may contribute to a more intense or longer activation of the healing cascade possibly resulting in the development of more adhesions, especially in neonates with pulmonary hypertension as explained in detail above.

### ECMO Therapy

To our knowledge, there is only one study explicitly reporting on SBO in correlation to ECMO therapy in patients with CDH. A protective effect of ECMO treatment was described with a significantly reduced rate of SBO of 9% in 22 patients with ECMO compared to 22% in 90 patients without ECMO (OR adjusted 0.2; 95% CI 0.0–1.0; *p* < 0.05) with no specification of the underlying cause of SBO (24). These findings could not be confirmed in our larger cohort regarding ASBO: a lower incidence was detected irrespective of ECMO therapy (6.9% in 130 patients with ECMO vs. 4.3% in 186 patients without ECMO, *p* = 0.32). Albeit a significant difference in ECMO treatment between both study cohorts (Janssen 19.6% vs. our cohort 41.1%; *p* < 0.0001), which may also have attributed to the different results. Possibly, the more relevant difference between both study cohorts is the timing of CDH repair: While Janssen et al. routinely perform CDH repair under ECMO therapy, we prefer to operate after weaning off ECMO. Therefore, alterations of the healing cascade, coagulation, and immune system due to using an ECMO circuit with cannulas, tubes, pumps, and blood from donors as well as specific drug administration and heparinization under ECMO therapy might play a role in the formation of adhesions and thereby explain the different findings in these two cohorts. Further basic research is needed to elucidate the biochemical and cellular processes involved and larger cohort studies with longitudinal follow-up to verify these findings.

### Time to Full Enteral Feeding

A correlation between time to enteral feeding and the occurrence of ASBO has been described in the literature. In neonates with spontaneous intestinal perforation, the duration of parenteral nutrition showed a significant effect on developing SBO later in life. However, a causal relationship was not confirmed by the authors but was considered to reflect the initial severity of the bowel disease (56). Regarding postoperative nutrition, neither the type nor the timing of postoperative oral feeding revealed a correlation with SBO in our cohort. Time to full enteral feeding showed a significantly longer time in the ASBO as compared to the non-SBO-group, but further analysis using logistic regression could not confirm these results. Therefore, it cannot be considered predictive of ASBO in our cohort. We started giving glucose on the first and breast milk or formula on the second postoperative day. Time to full enteral nutrition may be prolonged in children with more severe lung hypoplasia and the need for prolonged ventilatory support with consequently a longer time of analgosedation. This medication may also influence intestinal peristalsis with reduced intestinal motility and gastroesophageal reflux delaying enteral feeding and prolonging the need for parenteral nutrition. Reduced intestinal peristalsis might contribute to the formation of more dense or extensive adhesions. In our cohort, we could not detect an influence of any of the above-mentioned parameters, which may be due to the overall CDH severity in our open surgery cohort and different at other centers.

### Chest Tube

The duration of the chest tube insertion revealed a significant independent effect on the occurrence of SBO and ASBO. To the best of our knowledge, a correlation between the duration of the chest tube insertion and adhesive intestinal obstruction has not been described before. This circumstance may be explained in context with the specific population of patients with CDH, in which the separation between the thoracic and abdominal cavity is incomplete. Even after reconstruction, the diaphragm cannot be assumed impermeable, neither after primary nor after patch repair. Therefore, intrathoracic processes, such as chest tube insertion, could affect the abdominal environment. On the one hand, irritations of the tube induce a local inflammatory response and on the other hand, the injury of the pleura activates or prolongs physiological tissue repair processes (57).

Considering the correlation between the duration of the chest tube insertion and the type of pleural effusion, the influence of serous effusion or chylothorax or rather their consequences also have to be considered. Loss of chyle and its components, especially chylomicrons, proteins, and lymphocytes, lead to malnutrition, increased risk of thrombosis, and secondary immunodeficiency (58). In addition to the influence on the immune system, a procoagulant effect may also play a role in the formation of adhesions in the presence of chylothorax and substitution with fresh frozen plasma. An impaired flow of abdominal chyle might also be a causative factor for adhesion formation. Also, serous effusion is due to pleural and/or peritoneal injury and contains the so-called “reactive” mesothelial cells, macrophages and blood-derived cells like lymphocytes, and neutrophil granulocytes among others. “Reactive” mesothelial cells also display phagocytic activity. As has been explained above, these cells, tissue-factors, and inflammatory cytokines are involved in the pathogenesis of adhesion formation (7, 44). This might explain the correlation between serous effusion and chylothorax observed in our ASBO cohort, despite lacking significance for chylothorax due to the small patient number.

### Re-operations

In general, it is believed that repeated abdominal surgical interventions also trigger the formation of more adhesions. In children, this seems to be supported by a recurrence rate of ASBO from 0–29% (16). In a large study with a long follow-up of 500 adult patients with ASBO and also including those with surgical interventions in childhood, an increasing 10-year risk of obstruction was calculated in correlation with the number of episodes of ASBO: it was 18% after one episode and 63% after 4 episodes (59).

In contrast, re-operation was associated with a decreased rather than an increased risk of subsequent ASBO in our cohort. Yet, long-term follow-up has to be waited for the verification of these preliminary findings and final interpretation. In adults, similar findings were also reported. Patients with one previous surgery had more severe adhesions than patients with two surgeries, measured by the need for surgery for postoperative adhesive intestinal obstruction (40). Another study found that adults treated surgically for ASBO were less likely to have recurrent ASBO and the time to recurrence was longer. The authors indicated that in some of the patients treated surgically, the causative factor for adhesion formation was eliminated at the time of surgery (60). It must also be considered that in patients, who required laparotomy in the neonatal period, the incidence of SBO is higher than compared to older children (19, 22). This may be related to the reduced anti-inflammatory capacity and increased production of proinflammatory cytokines in neonates, which supports the persistence of adhesive formation, as described above (7–9). Our study participants, who underwent re-operation in the abdominal cavity other than for SBO, also received lysis of adhesions, and the timing of re-operation was mostly beyond the neonatal period. Presumably, a more mature immunological status at re-operation may lead to less adhesion formation and fewer sequelae.

### Mortality and Morbidity

In a review on adhesions in children and adolescents, mortality resulting from ASBO has been found to be 0% to anecdotal 71%, but nowadays, it is still correlated to septicaemia or the underlying comorbidities (16). In patients with CDH, an overall late mortality of 5% has been observed mainly with gastrointestinal complications (14). Though we did not observe mortality in our patients with SBO, there might be hidden mortality: two patients deceased at an older age due to severe “gastroenteritis”. Since no autopsy was performed, the real cause remains unknown but might as well have been due to either CDH recurrence with bowel incarceration or decompensated ASBO with consecutive septicaemia.

The need for bowel resection due to ASBO has been reported in 16% (17) and as much as 35% of patients after abdominal wall defects (18). In a large study of 414 neonates, intestinal perforation and gangrenous bowel were noted in 12.5 and 16.7%, respectively (15). Thus, the need for bowel resection in 17.6% of our patients is in accordance with the reports from the literature. Lautz et al. determined a delay of surgical intervention of >2 days after admission in patients without clinical improvement predictive of bowel ischemia and necrosis (61).

In children, a failure of conservative treatment of ASBO has been reported in 45–100%, while the majority of symptoms in adulthood resolve spontaneously, resulting in a much higher proportion of patients requiring relaparotomy in childhood (16). Accordingly, only three of our 38 patients with SBO (7.9%) could be managed conservatively in our cohort. These special findings should be considered in patients with CDH presenting with ASBO to reduce morbidity and mortality with a timely approach.

## Limitations and Strengths

First, this is not a multicentre study, but the follow-up of a large monocentric patient cohort treated with standardized surgical techniques and prospective follow-up may also be considered a strength. The high number of patients participating in our follow-up program after discharge may be another strength allowing for reliable detection of the incidence of SBO and aiming at identifying possible risk factors. Yet, the impact of adhesions is under-reported because symptoms due to adhesions that did not result in SBO were not included. Furthermore, the number of patients with a minimal-invasive repair is lower than the number of patients with an open approach. Neither the comparison of ASBO rate depending on different patch materials was possible due to the sole use of GoreTex® in this cohort nor was the comparison depending on different abdominal approaches because being an ECMO Center, a median laparotomy is the preferred access in our center for severely affected CDH neonates necessitating the implantation of an AWP in a substantial subset of patients.

## Conclusion

Symptoms of SBO are encountered with several underlying causes. Adhesive SBO was only observed after open CDH repair in our large cohort with prospective follow-up, which seems to underline the protective effect of MIS in a select subset of patients. In comparison to literature reports, a midline laparotomy might be associated with less ASBO than subcostal incisions. Furthermore, the implantation of Goretex® patches seems to be associated with less formation of adhesions, which is reflected by our comparatively low ASBO rate. Multivariate analysis revealed the duration of chest tube insertion as a risk factor and one or more re-operations as a protective factor for the occurrence of ASBO. Chest tube irritation over a prolonged period, possibly in combination with the cellular and immunologic consequences from serous effusion or chylothorax, may influence the occurrence of ASBO in patients with CDH during the neonatal period, reflecting an imbalance between anti- and proinflammatory responses. In the future, novel therapeutic strategies based on a better understanding of the biochemical and cellular processes involved in the pathophysiology of adhesion formation might contribute to a reduction of peritoneal adhesions and their associated morbidity and mortality.

## Data Availability Statement

The datasets presented in this article are not readily available because data was pseudonomized due to longitudinal follow-up and is saved in a local database. Requests to access the datasets should be directed to sylvia.buettner@medma.uni-heidelberg.de.

## Ethics Statement

The studies involving human participants were reviewed and approved by Ethics Committee II of the University of Heidelberg, Medical Faculty Mannheim. Written informed consent to participate in this study was provided by the participants' legal guardian/next of kin.

## Author Contributions

KZ and A-MF had full access to all the data in the study, take responsibility for the integrity of the data and the accuracy of the data analysis, study design, conduct, data collection, data analysis, and data interpretation, writing, and revision of this manuscript. TS, NR, MB, and LW were involved in the supervision of data collection, data interpretation, revision of the manuscript, and final approval. SB contributed to the statistical plan, data analysis, and critical revision of the manuscript. All authors contributed to the article and approved the submitted version.

## Conflict of Interest

LW received personal fees for his work in the Advisory Board Shire (intestinal failure in children due to short bowel syndrome) and for his lecture on pediatric surgery in neonatology (Chiesi). The remaining authors declare that the research was conducted in the absence of any commercial or financial relationships that could be construed as a potential conflict of interest.

## Publisher's Note

All claims expressed in this article are solely those of the authors and do not necessarily represent those of their affiliated organizations, or those of the publisher, the editors and the reviewers. Any product that may be evaluated in this article, or claim that may be made by its manufacturer, is not guaranteed or endorsed by the publisher.
